# Should we consider Dupuytren's contracture as work-related? A review and meta-analysis of an old debate

**DOI:** 10.1186/1471-2474-12-96

**Published:** 2011-05-16

**Authors:** Alexis Descatha, Pénélope Jauffret, Jean-François Chastang, Yves Roquelaure, Annette Leclerc

**Affiliations:** 1 Inserm U1018, Centre for Research in Epidemiology and Population Health, Epidemiology of occupational and social determinants of health, Villejuif, France; 2 Université de Versailles St-Quentin, UMRS 1018, France; 3 AP-HP, Poincaré University Hospital, Occupational Health Unit, Garches, France; 4 Laboratory of Ergonomics and Epidemiology in Occupational Health, University of Angers, Angers, France

**Keywords:** Dupuytren contracture, meta-analysis, observational studies, occupational

## Abstract

**Background:**

In view of the conflicting opinions published, a meta-analysis was undertaken on epidemiological studies in order to assess any association between Dupuytren's contracture and work exposure.

**Methods:**

Using the key words: "occupational disease", "work" and "Dupuytren contracture" without limitation on language or year of publication, epidemiological studies were selected from four databases (Pub-Med, Embase, Web of science, BDSP) after two rounds (valid control group, valid work exposure). A quality assessment list was constructed and used to isolate papers with high quality methodological criteria (scores of 13 or above, HQMC). Relevant associations between manual work, vibration exposure (at work) and Dupuytren's contracture were extracted from the articles and a metarisk calculated using the generic variance approach (meta-odds ratios, meta-OR).

**Results:**

From 1951 to 2007, 14 epidemiological studies (including 2 cohort studies, 3 case-control studies, and 9 cross-sectional studies/population surveys) were included. Two different results could be extracted from five studies (based on different types of exposure), leading to 19 results, 12 for manual work (9 studies), and 7 for vibration exposure (5 studies). Six studies met the HQMC, yielding 9 results, 5 for manual work and 4 for vibration exposure. Five studies found a dose-response relationship. The meta-OR for manual work was 2.02[1.57;2.60] (HQMC studies only: 2.01[1.51;2.66]), and the meta-OR for vibration exposure was 2.88 [1.36;6.07] (HQMC studies only: 2.14[1.59;2.88]).

**Conclusion:**

These results support the hypothesis of an association between high levels of work exposure (manual work and vibration exposure) and Dupuytren's contracture in certain cases.

## Background

Dupuytren's contracture is characterized by chronic contracture of the fourth and fifth fingers of the hand toward the palm, usually accompanied by thickening of the palmar skin [1-3]. Prevalence rates range from 0.2% to 56% in various age and population groups, and methods of data collection [4]. In his presentation on December 5, 1831, at the Hotel-Dieu in Paris, Baron Guillaume Dupuytren clearly identified the main lesion of the disorder as contracture of the palmar fascia, which he asserted could be surgically treated by excision of the palmar aponeurosis [5]. In that lecture, Baron Dupuytren associated the disease with chronic local trauma caused by occupation [6]. "Most people with this disease have been obliged to do work with the palm of the hand or to handle hard objects. Thus the wine merchant and the coachman whose case histories we will report were accustomed, one to broaching casks with a puncheon or to binding up staves, the other to plying his whip unceasingly on the backs of his jaded horses. We could also cite the example of a clerk in an office who took particular care in applying the seal to his dispatches. It is also found in masons who grasp stones with the end of their fingers,[...]. For this it is clear that the disease affects particularly those who are obliged in their work to use the palm of their hand as a pressure point." Previously, Henry Cline, Sr., a prominent London physician, recognized the disease in 1787 as one contracted by "laborious people" [6]. In 1822, Sir Asteley Cooper attributed the contracture to "excessive action of the hand, in the use of the hammer, the oar ...".

Although there is general consensus concerning certain genetic predisposing factors [7] and other risk factors such as diabetes, smoking and alcohol intake (with discussion about epilepsy/anticonvulsant drugs) [3,8], the apparently conflicting results regarding the possible work-related origin of this disease are still a subject of debate [9,10]. A systematic review to address this controversy in 1996 concluded that there is good support for an association between vibration exposure and Dupuytren's contracture, and a weaker association with manual work (5 studies but only one met the criteria suggested by the authors for methodological quality) [11]. The authors suggested then that further studies are needed with better characterization of exposure in that area, and highlighted the prevention consequences for workers and ergonomists/occupational practitioners.

However, since this comprehensive review, occupational exposure and vibration have not been considered by many clinicians as potential risk factors for Dupuytren's contracture [2,4,12], although additional studies published in the last ten years have supported an association between work exposure (manual work and vibration) and Dupuytren's contracture [13,14].

The aim of this study was to undertake a systematic review and meta-analysis of the available epidemiological data regarding the association between work exposure (manual work and vibration exposure) and Dupuytren's contracture.

## Methods

### Literature research

Four databases (Pub-Med, Embase, Web of science, "Base de Données de Santé Publique", BDSP, i.e. the French Public Health Database,) were searched by using the key words: "occupational disease", "work" and "Dupuytren contracture". No language limitation was added. Interesting papers originating from the reference list of full-text papers and reviews were also included at this stage. The first selection of articles was performed by two independent readers based on the title and abstract to include only (i) original epidemiological studies (with control group, case series not included), for which (ii) the association between manual work (either heavy manual labor or exposure to vibrations) and Dupuytren's contracture was reported, with occupational exposure clearly described (exposure defined or at least discussed). The second stage included full-text papers, based on the same criteria, and only studies meeting these criteria were included in the meta-analysis after review by the independent readers (A.D. and P.J.).

### Assessment of methodological quality

A quality assessment list was constructed using criteria from the Cochrane Centre, and recent reviews on musculoskeletal disorders at work [15,16], adapted to Dupuytren's contracture. The list comprised five topics covering 20 items in total: i.e. study population, assessment of exposure, assessment of outcome, study design and analysis and data presentation (Appendix 1- Additional file 1,). Two reviewers (A.D. and P.J.) independently assessed the quality of each study by scoring each criterion as positive or negative. Disagreement was resolved by consensus. The quality score for each study was calculated by adding together the number of positive criteria. The high quality methodological study criterion was based on a total score of 13 or higher. The threshold was chosen to represent over two-thirds of the scale.

### Data extraction and analysis

Relevant data were extracted from the articles. The core findings in each article were expressed by measures of association (odds ratio) with corresponding 95% confidence interval (CI). When possible, such associations were directly extracted from the original article (with adjustments if available). In articles where this information was not presented, associations were calculated if sufficient raw data was provided and in some cases by contacting the authors. If two OR were presented in the study and if they concerned different exposures/populations, both were included. However, if the exposure was similar, only the OR related to the most precise exposure, higher dose and/or adjusted model was included.

Results were treated as all work exposure together, then divided into manual work and vibration exposure. Metarisks (meta-odds ratios, meta-OR) were also run only on high quality methodological studies in each exposure sub-group.

Meta-ORs were calculated using the generic variance approach. The weight given to each study is the inverse of the variance of the estimated effect. Heterogeneity was tested with the Q statistic. From the Q statistic, we calculated summary OR and 95% CI with the random effect method [17]. This approach provides more conservative estimates (wider CI) than a fixed effect model, assuming that the differences between results are solely due to chance. We explored publication bias due to study size by drawing Funnel plots and testing with Egger's regression approach.

The meta-analysis was performed using STATA (Version 10.0 ; Stata Corp., College Station, TX, USA). The MOOSE and PRISMA checklists were used (Appendix 2: Additional file 2) [18,19].

## Results

**Figure 1:**
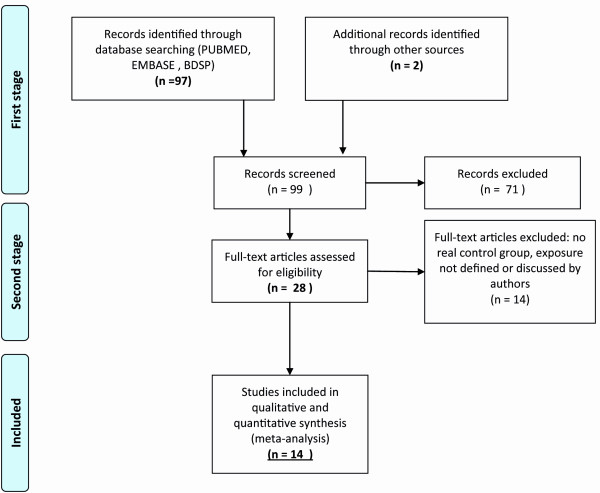
**Flow diagram (adapted from **[19]).

We found 99 papers in the four databases corresponding to our first stage, and 28 papers were included and scored blind after reading the abstracts and titles and using cross references (second stage, Figure 1). After full-text reading, four papers not related to work exposure and 10 papers that were not methodologically appropriate were excluded (no real control group, [20-26] exposure not defined or discussed by authors) [27-29].

Table 1 presents the 14 papers selected for the meta-analysis (10 in English, 2 in French, 1 in Italian, 1 in German) [13,14,30-41]. The studies originated only from European countries, mostly Northern Europe (one in North and Central Italy, one in Sardinia), and were published from 1951 to 2007 (6 studies published since the review of Liss and Stock in 1996) [11]. Cross-sectional design and population survey were found in 9 studies of 14 (3 case-control and 2 cohort studies). Clinical examination was the diagnostic method for all studies. Exposure was assessed differently, including job title, self-reported exposure and measurements (for vibration exposure). Two different results could be extracted from five studies, as they were based on different types of exposure: based on different groups of exposed jobs [13,32,38], different populations [36], or a particular subgroup with different types of work exposure, manual work and vibration [14]. After contact with the authors, overall biomechanical exposure ("all") included: using a tool with a handle or a vibrating tool, manual handling and repairing mechanical equipment. For the combined meta-OR of vibration (using a vibrating tool) and manual work (using a tool with a handle, manual handling and repairing mechanical equipment) were considered separately.

**Table 1:** Papers selected in the final round

Name	Country	Type of study	Outcome	Exposure	Study population: exposure	Patients with Dupuytren's Contracture	Work Exposure?	Score	Criteria for Odds Ratios (OR)	OR			Major Strength(s)	Major limitation(s)
**Bennnet 1982**	United Kingdom	Cross sectional	Physical examination (inspection, scheme and chart)	Job title and precise questionnaire	216 workers in PVC bagging and 84 others	17 (16 in bagging -1 in the control group)	Manual work	14*	Bagging plant vs non-bagging plant	5.5	0.8	36.7	Confounders	Exposure imprecise
**Gudmundsson 2000 (1)**	Iceland	Cohort	Physical examination (two stages of severity)	Self-administered questionnaire, checked with specially trained secretary (Reykjavik Study)	1297 men including 128 manual workers and 126 tradesmen	249 (including 38 in manual labor, 36 tradesmen)	Manual work	16*	Manual labor (seamen. farmers) vs controls	1,75	1,14	2,7	Cohort, confounders	Exposure assessment
**Gudmundsson 2000 (2)**	Iceland	Cohort	Physical examination (two stages of severity)	Self-administered questionnaire, checked with specially trained secretary (Reykjavik Study)	1297 men including 128 manual workers and 126 tradesmen	249 (including 38 in manual labor, 36 tradesmen)	Manual work	16*	Skilled trades (masons. carpenters, blacksmith) vs controls	1,91	1,24	2,96	Cohort, confounders	Exposure assessment
**Godtfredsen 2004**	Denmark	Cohort	Physical examination (trained nurses or MD student)	Self-administered questionnaire in a large study (Copenhagen City Heart Study)	7254 participants, 2923 low education ** (280 highly physical job)	772	Manual work	14*	Low education ** level (considered as a proxy for manual labor) vs high	1.6	1.22	2.1	Cohort, confounders	Exposure assessment considered as a proxy for manual work
**Lucas 2008 (1)**	France	Cross sectional	Physical examination (occupational physician)	Detailed interview	2406 men working for the equipment ministry (643 highly exposed to force, and 350 highly exposed to vibrations)	212 (including 106 in high exposure group and 47 in high vibration group)	Manual work	14*	High cumulative work exposure vs low ***	3.1	1.99	4.84	Exposure, dose -response relationship, confounders and study of interactions	Cross sectional, smoking missing, included manual work
**Herzog 1951 (1)**	United Kingdom	Cross sectional	Physical examination (by the author only)	Job title but individual visit to works and offices	503 steelworkers (men over 40 years), 451 miners (men over 40 years), and 480 clerks (men over 40 years, controls)	61 (22 steelworkers and 21 miners)	Manual work	6	Steelworkers vs clerical	1.2	0.6	2.3	First large published epidemiological study	Exposure assessment, confounders
**Herzog 1951 (2)**	United Kingdom	Cross sectional	Physical examination (by the author only)	Job title but individual visit to works and offices	503 steelworkers (men over 40 years), 451 miners (men over 40 years), and 480 clerks (men over 40 years, controls)	61 (22 steelworkers and 21 miners)	Manual work	6	Miners vs clerical	1.3	0.6	2.5	First large published epidemiological study	Exposure assessment, confounders
**Early 1962**	United Kingdom	Cross sectional	Physical examination (inspection, palpation, system of staging described)	Job title in similar workplace (office vs locomotive works)	4454 manual workers at locomotive works and 423 male office workers (<65 years)	151 (134 in Crewe locomotive works with manual work, 17 in office)	Manual work	7	Manual vs clerical	0.98	0.6	1.7	Large sample	Exposure assessment, confounders
**Mikkelsen 1978**	Norway	Population survey	Physical examination with a staging scheme	From records of occupation, different levels of exposure assessed by interview	6888 men (including 477 with heavy manual work) and 4120 women (including 6 with heavy manual work)	647 men with DC (including 70 in heavy manual work) and 254 women with DC (including 1 in heavy manual group)	Manual work	11	Heavy work vs light**** (men and women)	3.1	2.2	4.4	Dose -response relationship (severity and exposure)	Except for age, no confounders taken into account, and no duration of exposure
**Attali 1987**	France	Cross sectional	Physical examination by gastroenterologist (three stages of severity)	Detailed interview	432 patients- 258 with liver disorders and 174 controls, 42.1% of these being manual workers	78 (56 with liver disease and 22 controls)	Manual work	10	Manual workers	2.46	1.49	4.06	Large number of cases	Exposure assessment, confounders
**Niezborala 1995 (1)**	France	Case-control	Physical examination	Precise questionnaire	227 patients including 43 with high forceful work in their longest job	121 (including 29 in the high exposure group)	Manual work	12	Case control study (masons and lumberjacks vs others, longest job)	2.41	1.18	4.92	Information on length of exposure, confounders	Statistical analyses used for confounders
**Niezborala 1995 (2)**	France	Cross sectional	Physical examination (and severity score)	Precise questionnaire	324 workers, with 191 builders or farmers and 133 non-manual work	31 (including 28 in the exposed group)	Manual work	11	Cross sectional study (exposed = builders and farmers vs others)	7.5	2.21	24.7	Information on length of exposure, confounders	Statistical analyses used for confounders
**Cocco 1987**	Italy	Case-control	Physical examination (definite contracture only)	Detailed interview	14557 patients from Occupational health institute, 80 workers with >20 years of vibration exposure; 150 non-exposed	180 (paired with 180 controls on sex, age, date of hospitalization)	Vibration exposure	14*	>20 years of exposure vs controls	3	1.3	6.7	Case control study, dose -response relationship, exposure information	Confounder analysis
**Bovenzi 1994 (1)**	Italy	Cross sectional	Physical examination (no detail)	Detailed interview and measurement of vibration levels	145 quarry-drillers and 425 stone carvers, 258 controls	66 (57 in workers group, 9 controls)	Vibration exposure	14*	Quarry-drillers vs controls	2.58	1.07	6.2	Dose -response relationship, exposure information, confounder analysis	Cross sectional design
**Bovenzi 1994 (2)**	Italy	Cross sectional	Physical examination (no detail)	Detailed interview and measurement of vibration levels	145 quarry-drillers and 425 stone-carvers, 258 controls	66 (57 in workers group, 9 controls)	Vibration exposure	14*	Masons and stone-carvers vs controls	2.6	1.24	5.49	Dose -response relationship, exposure information, confounder analysis	Cross sectional design
**Lucas 2008 (2)**	France	Cross sectional	Physical examination (occupational physician)	Detailed interview	2406 men working for the equipment ministry (643 highly exposed to force, and 350 highly exposed to vibrations)	212 (including 106 in high exposure group and 47 in high vibration group)	Vibration exposure	14*	High cumulative vibration exposure vs low***	1.82	1.24	2.68	Exposure, dose -response relationship, confounders and study of interaction	Cross sectional, lack of blindness, smoking missing
**Chanut 1963**	France	Cross sectional	Physical examination (inspection, palpation, system of staging described)	Detailed interview	180 stonemasons, 13500 clerks	378 (25 stonemasons, 130 clerks and 223 others)	Vibration exposure	10	Stone masons vs others	14.57	9.53	22.51	Clinical details	Exposure assessment, confounders
**Thomas 1992**	United Kingdom	Cross sectional	Physical examination (no detail)	Detailed interview	311 claimants considered to have Vibration white fingers and aged from 50-85 years (and considered as exposed to vibration) and 150 hospital control group	78 (62 in the exposed group)	Vibration exposure	6	Vibration-exposed vs hospital admission	2.1	1.1	3.9	Dose -response relationship, duration of exposure)	Confounders analysis and selected case for dose-response relationship
**Seidler 2001**	Germany	Case-control	Physical examination (hand surgery center)	Detailed interview	Cases from two clinics, with 33 males exposed to vibration (over 20 h/week and over 20 years)	317 (including 17 exposed to vibration > 20 h/week and over 20 years)	Vibration exposure	12	>20 h/week over 20 years of vibration	1.3	0.6	2.7	Confounders and different job exposure	Selection bias, exposure assessment

*: met the high quality methodological study criterion (score of 13 or above).

**: low level of education was defined as <8 years of school education, and high level as ≥12 years

***: level of exposure was based on a cumulative score including number of years of manual work for each task considered (using a tool with a handle or a vibrating tool, manual handling, and repairing mechanical equipment) and the average annual frequency. The total score obtained was divided into three categories (low, medium and high exposure)

****: heavy work was for instance lumberjacks, full time farmers; light manual work (or none), dentists, clerks, vicars.

Six studies met the high methodological quality criteria (≥13/20, 9 results, good agreement between the two readers, >90%). Five studies reported a clear dose-response relationship (higher exposure corresponding to higher OR or more severe disorder), whereas one did not, but this sample included only workers with vibration white finger syndrome [40].

**Figure 2:**
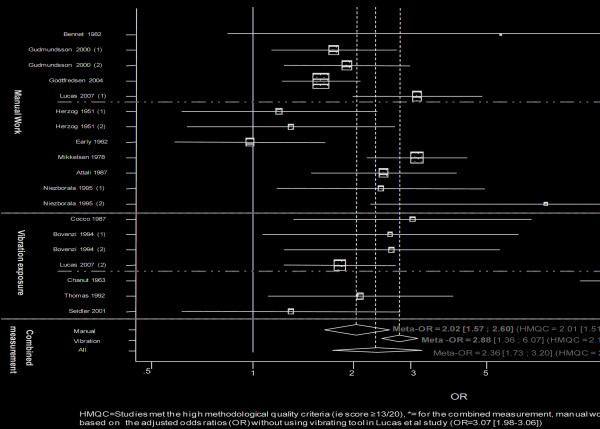
**Forest plot**. The black square and horizontal line correspond to the studies' odds ratios (OR) and 95% confidence intervals. The area of the black squares reflects the weight each study contributes to the meta-analysis. The diamond represents the meta-OR with its 95% confidence interval.

The overall meta-OR was significantly higher than 1 (Figure 2): the meta-OR for manual work was 2.02 [1.57;2.60], and the meta-OR for vibration at work was 2.88 [1.36;6.07]. The meta-OR calculated from the studies which met the high methodological quality criteria was similar to the meta-OR of all studies (2.01 [1.51; 2.66] and 2.14 [1.59;2.88] for manual work and vibration exposure, respectively). Funnel plot and Egger's test did not suggest a major publication bias.

## Discussion

The results of this meta-analysis support the hypothesis of an association between high work exposure, manual work and exposure to vibration, and Dupuytren's contracture in certain cases.

There may have been a publication bias, although we feel it was not an issue here. Indeed, negative studies have been published and were included in our initial list of selected papers [30,32,41] and in the second round of selection [20,23,26-29]. Egger's test and funnel plot did not reveal publication bias. The methodology used to select papers and extract data from them may also have induced a bias. Blind reviewing with scoring helped to reduce this effect, especially with the good agreement between the two readers. The choice of the OR used in the meta-analysis may have been inappropriate in cases of high numbers of results, but this seemed to be a minor problem in this review because of the similarity of the results, except for the study by Godtfredsen et al [31]. The education variable was considered be compatible with the authors' choice instead of physical activity at work to when it was included in their last adjusted model (considering that low educational level is strongly correlated with manual work and hence a a proxy for it). Out of the 10 papers not selected because of major limitations, four were positive. Another strong element supporting validity was comparison with the 1996 review by Liss and Stock [11]. Although the criteria used were different (selection and quality scoring), there was a good overlap between studies (before 1996) which met their high methodological quality criteria and those presented here: of the four studies meeting their validity criteria [30,37,38,40], three of them met our high methodological quality criteria [30,37,38], and no other high quality paper published before 1996 was selected for our study.

It is also necessary to consider study design since only two cohort studies were found. In cross sectional studies, workers with Dupuytren's contracture may be more likely to describe their work as strenuous. However, studies were selected on the basis of exposure provided with relative precision (in order to limit any potential recall bias) and one on vibration measurements [38]. Clinical assessment was used in all of the studies retained, because this is considered to be the gold standard for Dupuytren's contracture [2], with a good agreement between clinicians (kappa statistic from 0.7 to 1.0) [42]. When the differences between negative and positive evidence on associations between occupational exposure and Dupuytren's contracture were examined, the main difference observed was exposure quantification: "manual work" appears to be not sufficiently precise to be related to Dupuytren's contracture, which probably explains why many studies based only on job title were found to be negative in large populations with heterogeneous levels of exposure [29,32,33].

This meta-analysis showed that high cumulative work exposure (intensity × duration) was associated with Dupuytren's contracture. Manual work and vibration exposure are closely related in many jobs [14]. The dose-response relationship found in 5 publications supports this association. The lack of dose-response reported by Thomas *et al *was possibly due to selection bias, with subjects highly exposed to vibration (enough to have vibration white finger syndrome) [40]. Dupuytren's contracture is currently considered to be a fibroproliferative disorder, with dysfunction of connective tissue and fibroblast proliferation. Although the cause and pathophysiology are still the subjects of much research, many elements have recently been discovered [1]. The roles of high levels of repetitive strain and vibration exposure are plausible, especially as a result of the local hypoxia and chronic ischemia hypothesized in Dupuytren's contracture [8,43]. All the studies originated from Europe, mostly Northern Europe, probably because the prevalence is higher there than elsewhere. There is also probably genetic susceptibility to the disease [7,8,28]. However, a genetic predilection does not modify the consistency of the results and the conclusions, as discussed by Niezborala et al [36], or the lack of interaction between work exposure and familial history of Dupuytren's contracture found in Lucas et al's study [14]. Similar magnitudes of strength of association found in the different studies presented reinforced the plausibility of a causal relationship.

## Conclusion

The conclusion of this meta-analysis is that high cumulative exposure to physical constraints in terms of force and/or vibrations transmitted to the upper limbs was associated with the occurrence of Dupuytren's contracture, at least in European countries, confirming and reinforcing the review of Liss and Stock. Work compensation in some cases with documented high levels of exposure and the few other risk factors should therefore be discussed and in some cases awarded. In each case of Dupuytren's contracture case, the occupational practitioner should discuss improvements in working conditions with ergonomists, in order to slow the evolution of the disorder and/or its consequences or at least prevent new cases in workers with similar tasks. Long-term longitudinal studies on large samples with valid exposure, taking into account the effects of interactions with other risk factors, would be valuable.

## List of abbreviations

OR: odds ratio; HQMC: high quality methodological criterion (figure).

## Competing interests

The authors declare that they have no competing interests.

## Authors' contributions

A Descatha designed the study, and participated in data collection, data interpretation and writing. P Jauffret participated in data collection, data interpretation, commenting on the manuscript and improving the English. JF Chastang performed the analyses and constructed figures and participated in commenting on the manuscript. Y Roquelaure and A Leclerc participated in the development of the study, data interpretation, and commenting on the manuscript.

All authors read and approved the final manuscript.

## Authors' information

The authors are members of research units in occupational health and A Descatha, Y Roquelaure and A Leclerc are members of the Musculoskeletal Committee of the International Commission of Occupational Health (ICOH), and the French Language Research group on MSD.

## Pre-publication history

The pre-publication history for this paper can be accessed here:

http://www.biomedcentral.com/1471-2474/12/96/prepub

## Supplementary Material

Additional file 1 **Appendix 1. Quality assessment list used**. The quality assessment list used was constructed using criteria from the Cochrane Centre, and recent reviews on musculoskeletal disorders at work [15,16] adapted to Dupuytren's contracture. Click here for file

Additional file 2 **Appendix 2. PRISMA AND MOOSE Checklists**. The meta-analyses quality checklist (adapted from [18,19]). Click here for file
