# Supplementary material for: Should we consider Dupuytren's contracture as work-related? A review and meta-analysis of an old debate
Source: BMC Musculoskelet Disord. 2011 May 16;12:96. doi: 10.1186/1471-2474-12-96 (PMC3123614; doi:10.1186/1471-2474-12-96)
Supplement: Additional file 1 — Appendix 1. Quality assessment list used. The quality assessment list used was constructed using criteria from the Cochrane Centre, and recent reviews on musculoskeletal disorders at work [15,16] adapted to Dupuytren's contracture. [file 1471-2474-12-96-S1.DOC]

# Appendix:

Quality assessment list used.

|  | Criteria and attribution of points |
| --- | --- |
| **STUDY POPULATION**  Positive if items in both groups were reported at baseline :   1. Age (mean (SD or CI), or dichotomised groups) and gender 2. Alcohol and/ or smoking 3. Heredity and/ or diabetes and/ or epilepsy   Positive if the participation of the exposed group and unexposed group was ≥ 80%  Positive if the total number of cases was ≥ 50  **ASSESSMENT OF EXPOSURE**  Adequate description of exposure   1. Positive if the exposure was clearly defined 2. Positive if the assessment of exposure was described 3. Positive if the exposure was assessed by an independent person and was not based on self-reported exposure   **ASSESSMENT OF OUTCOME**  Adequate description of the outcome   1. Positive if the outcome was clearly defined 2. Positive if the method of assessment was suitable 3. Positive if the outcome was measured without knowledge of the exposure status by an independent person, thus not based on self reported symptoms.   **STUDY DESIGN**  Positive if the study design was prospective or a retrospective cohort/case control  Positive if inclusion and exclusion criteria were described  Positive if the follow-up period was ≥ 1 year  Positive if personal information was given for completers and withdrawals  **ANALYSIS AND DATA PRESENTATION**  Positive if risk estimates were presented or when raw data were given which allow the calculation of risk estimates, such as: Odds ratios, prevalence ratios or relative risks  Identifying confounders (at least 2, in addition to age/gender)   1. Positive if the confounders considered were described 2. Positive if the method used to control for confounding was described | **1** = yes if both age/gender are reported **0** = else  **1** = yes if alcohol OR tobacco **0** = else  **2** = all three **0** = none **1**= else  **1** = yes **0** = no  **1** = yes **0** = no  **1** = yes **0** = no  **1** = yes **0** = no  **1** = yes **0** = no  **1** = yes **0** = no  **1** = yes **0** = no  **1** = yes **0** = no  **1** = yes **0** = no  **1** = yes **0** = no  **1** = yes **0** = no  **1** = yes **0** = no  **1** = yes **0** = no  **2** = if there were two **1**= if there was one **0**= none  **1** = yes **0** = no |
| **TOTAL** | **0 to 20** |

*Constructed using criteria from the Cochrane Centre, and recent reviews on musculoskeletal disorders at work [15,16] adapted to Dupuytren’s contracture.*
